# The effects of tolvaptan on patients with severe chronic kidney disease complicated by congestive heart failure

**DOI:** 10.1007/s10157-013-0788-6

**Published:** 2013-03-13

**Authors:** Tomoyuki Otsuka, Yukinao Sakai, Dai Ohno, Tsuneo Murasawa, Naoki Sato, Shuichi Tsuruoka

**Affiliations:** 1 Division of Nephrology, Department of Internal Medicine, Nippon Medical School Musashikosugi Hospital, 1-396 Kosugi-cho, Nakahara-ku, Kawasaki, 211-8533 Japan; 2 Division of Cardiology, Department of Internal Medicine, Nippon Medical School Musashikosugi Hospital, Kawasaki, Japan; 3 Division of Nephrology, Department of Internal Medicine, Graduate School of Medicine, Nippon Medical School, Tokyo, Japan

**Keywords:** Tolvaptan, Chronic kidney disease, Diuretic, Congestive heart failure, Renoprotection

## Abstract

**Background:**

Tolvaptan, a diuretic with a new mechanism of action, selectively binds to the vasopressin V2 receptor and inhibits reabsorption of water. Its effect on heart failure is proven, but its benefit for patients with chronic kidney disease (CKD) has not been not confirmed. In this study, we examined the effect of tolvaptan on patients with severe CKD.

**Methods:**

We analyzed patients with stage 4 or higher CKD who had congestive heart failure that was resistant to existing diuretics. The patients were administered an initial tolvaptan dose of 7.5 mg/day. We assumed urine volume and urine osmolality to be the main effective endpoint and recorded free water clearance, serum osmolality, serum creatinine (Cr) level, and adverse events.

**Results:**

There was no instance of clinically significant hypernatremia. The urine volume increased significantly (*P* < 0.0001), as did the urine osmolality (*P* = 0.0053). Free water clearance showed a tendency to increase, although the difference was not statistically significant. The serum creatinine level did not change significantly, and there was no clear effect on renal function. However, in patients with stage 5 CKD, the serum creatinine level decreased significantly (*n* = 5, *P* = 0.0435). There were no adverse events.

**Conclusion:**

We confirmed that tolvaptan has a diuretic effect in patients with both severe CKD and congestive heart failure without causing either clinically significant hypernatremia or an adverse effect on renal function. Tolvaptan is an effective diuretic for patients with CKD.

## Introduction

Tolvaptan binds selectively to the V2 receptor (1 of the 3 vasopressin receptors: V1a, V1b, and V2), disturbs the movement of aquaporin 2 into the luminal side of cortical collecting duct cells through activation of cAMP, and inhibits reabsorption of water. It thus uses a new mechanism of action for producing water diuresis [1, 2]. The effect of tolvaptan is expected to be unlike that of conventional diuretics [3], and its short-term effects for treating heart failure have been investigated in the ACTIVE in CHF [4] and EVEREST [5, 6] studies. However, careful administration has been suggested, because volume depletion by diuresis leads to a decrease in renal blood flow in patients with serious renal dysfunction; thus, renal function may worsen [7]. However, one study has suggested that the renal blood flow and glomerular filtration rate (GFR) are not reduced by tolvaptan [8]. In addition, the protective function of the kidney is expected to initiate a diuretic effect without activating the renin–angiotensin system [9, 10]. There are many unanswered questions about the effect of tolvaptan on renal function, and there are few reports of its use for patients with severe renal dysfunction [11]. In this report, we examined the effect of tolvaptan in patients with severe chronic kidney disease (CKD) complicated by congestive heart failure who were resistant to existing diuretics.

## Subjects and methods

This report is a retrospective observational study of usual practice, and there was no planned protocol. However, we explained the likelihood of side effects of tolvaptan, which was a new medicine, to all patients and obtained their consent. We included patients with stage 4 CKD or higher and congestive heart failure who were admitted to our hospital. The initial tolvaptan dose was 7.5 mg/day. After 2 or 3 days, the dose was increased to 15 mg/day depending on the observed efficacy and adverse events. The treatment-targeted value for serum Na concentration controls was set at 144 mEq/l. If the serum Na concentration increased to ≥145 mEq/l, we reduced the tolvaptan dose. Urine volume and urine osmolality were assumed to be the main effective endpoint. We evaluated free water clearance, serum osmolality, serum creatinine (Cr) level, and adverse events. In addition, we compared values of human atrial natriuretic peptide (HANP) and B-type natriuretic peptide (BNP) before the administration of tolvaptan and 1 month later.

The value of each measurement is expressed as mean ± standard deviation (SD). We conducted one-way analysis of variance (ANOVA) by considering data multiplicity over time and used Tukey’s multiple comparison test for the subsequent post hoc test. We used the paired *t* test for comparisons of HANP and BNP values. We considered *P* < 0.05 as statistically significant. In addition, for each set of data, a regression line was obtained.

## Results

Tables 1 and 2 show a summary of the patients’ backgrounds. The study group consisted of 5 men and 3 women with a mean age of 53.7 ± 7.7 years and a mean serum Cr level of 7.57 ± 5.66 mg/dl at admission. Their cardiac function grade was assessed according to the New York Heart Association (NYHA) criteria. Five patients were class II and 3 patients were class III. Primary diseases included rapidly progressive glomerulonephritis (*n* = 1), methicillin-resistant *Staphylococcus aureus*-associated nephritis (*n* = 1), benign nephrosclerosis (*n* = 1), polycystic kidney disease (*n* = 3), and diabetic nephropathy (*n* = 2). Patients were using the following diuretics: azosemide (60 mg/day; *n* = 1), eplerenone (50 mg/day; *n* = 1), torasemide (8 mg/day; *n* = 2), and furosemide (40–200 mg/day; *n* = 6). The renin-angiotensin-aldosterone system (RAAS) inhibitor (olmesartan) was prescribed for 7 patients at a dose of 40 mg. Eplerenone (50 mg) was prescribed for the remaining 1 patient. No patient took digitalis.

**Table 1:** Patient baseline characteristics (*N* = 8)

Parameter	Statistics
Blood pressure (mmHg)	
Systolic	155.3 ± 24.8
Diastolic	88.8 ± 17.9
NYHA II:III, *n*	5:3
HANP (pg/ml)	255.6 ± 236.5
BNP (pg/ml)	1012 ± 1356
sCr (mg/dl)	7.57 ± 5.66
sCr stage 5 (mg/dl)	10.08 ± 5.91
Na (mEq/l)	138.0 ± 6.3
UV (ml/day)	1263 ± 655
uOsm (mOsm/kg)	275.0 ± 39.8
sOsm (mOsm/kg)	296.5 ± 7.6

*BNP* B-type natriuretic peptide, *HANP* human atrial natriuretic peptide, *NYHA* New York Heart Association, *uOsm* urine osmolality, *sOsm* serum osmolality, *UV* urine volume

**Table 2:** Patient baseline profile (*N* = 8)

No.	Age	Gender	Primary diseases	CKD stage	NYHA	Tolvaptan (mg)	Furosemide (mg)	Torasemide (mg)	Azosemide (mg)	Eplerenon (mg)	Olmesartan (mg)
1	56	M	Nephrosclerosis	5	III	15	180				40
2	64	F	PKD	5	II	15	200				40
3	50	M	MRSA nephritis	5	III	7.5	120		60		40
4	49	M	PKD	5	II	7.5		8			40
5	65	F	PKD	5	II	7.5	140			50	
6	51	F	RPGN	4	II	15		8			40
7	53	M	DN	4	II	15	180				40
8	42	M	DN	4	III	15	40				40

*CKD* chronic kidney disease, *DN* diabetic nephropathy, *NYHA* New York Heart Association, *MRSA nephritis* methicillin-resistant *Staphylococcus aureus*-associated nephritis, *PKD* polycystic kidney disease

The dose of tolvaptan remained constant after the 3rd day, with 5 patients receiving 15 mg/day and 3 receiving 7.5 mg/day. During the course of the study, 1 patient’s Na concentration exceeded 145 mEq/l; however, this did not continue for more than 24 h and eventually decreased to <144 mEq/l. Therefore, we did not reduce the tolvaptan dose.

**Fig. 1:**
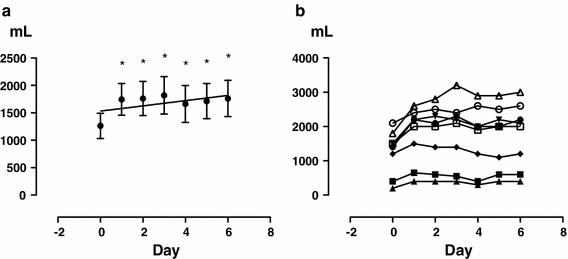
Overall changes in 24 h urine volume (**a**) and each change in each patient (**b**). *Significant according to the results of a one-way ANOVA (*P* < 0.0001) and Tukey’s multiple comparison testing (0 vs. 1, 0 vs. 2, 0 vs. 3, 0 vs. 4, 0 vs. 5, 0 vs. 6)

**Fig. 2:**
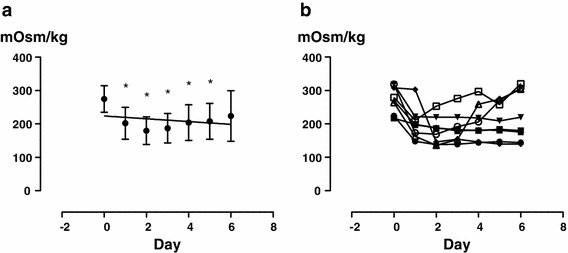
Overall changes in urine osmolality (**a**) and each change in each patient (**b**). *Significant according to the results of a one-way ANOVA (*P* = 0.0010) and Tukey’s multiple comparison testing (0 vs. 1, 0 vs. 2, 0 vs. 3, 0 vs. 4, 0 vs. 5)

**Fig. 3:**
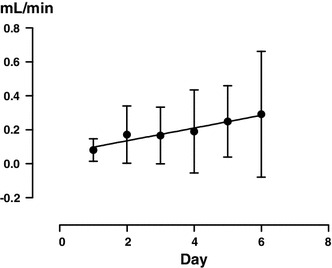
Changes in free water clearance

**Fig. 4:**
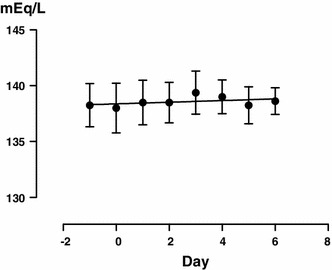
Changes in serum Na concentration

Urine volume increased (Fig. 1), with a significant difference from the next day (*P* < 0.0001), and the urine osmolality decreased similarly (Fig. 2) (*P* = 0.0010). Free water clearance showed a tendency to increase, but the difference was not significant (Fig. 3). The serum osmolality showed almost no change, as was the case for the serum Na concentration (Fig. 4).

**Fig. 5:**
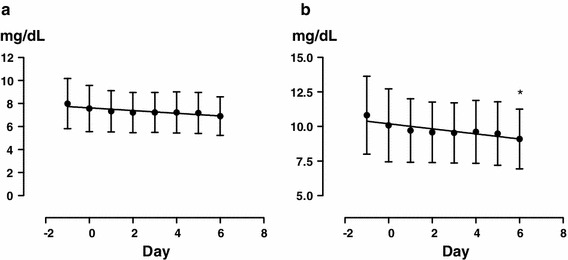
Overall changes in serum Cr level (**a**) and in stage 5 CKD patients alone (**b**). *Significant according to the results of a one-way ANOVA (*P* < 0.0435) and Tukey’s multiple comparison testing (0 vs. 6)

The serum Cr level did not show a significant change, and there was little effect on renal function (Fig. 5a). However, the serum creatinine level significantly decreased when it was analyzed for patients with CKD stage 5 alone (Fig. 5b) (*n* = 5, *P* = 0.0435).

**Fig. 6:**
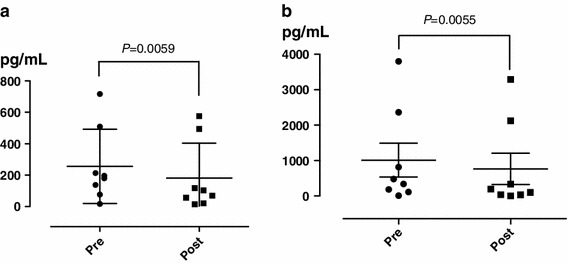
Changes in human atrial natriuretic peptide (HANP) (**a**) and B-type natriuretic peptide (BNP) (**b**). *P* values are compared with baseline using the paired *t* test

HANP and BNP decreased significantly (Fig. 6) (*P* = 0.0059 and 0.0055, respectively). However, blood pressure showed a tendency toward decreasing, but the difference was not significant (data not shown).

## Discussion

In this study, we showed that tolvaptan produced a consistent diuretic effect among patients with severe CKD and congestive heart failure. If the kidney has some residual renal function, tolvaptan, which is a water diuretic that significantly decreases urine osmolality, enables maintenance of the osmoregulation of the body fluids by the renal cortical collecting tubules. However, clinically significant hypernatremia did not occur, probably because we used a natriuretic in combination with tolvaptan. In addition, in accordance with alleviation of congestion by tolvaptan, the effect of furosemide may also be improved. This may be one of the reasons why the urine osmolality and urine volume did not change in parallel.

A study reported increased renal blood flow after administration of tolvaptan among patients with heart failure, but this finding was not observed among patients with renal failure [8]. The mechanism underlying this effect is not yet understood. One of the reasons for the improvement in the serum Cr level in CKD stage 5 patients may be increased renal blood flow with tolvaptan. Further, the serum Cr level may have decreased because “congestive kidney failure” [12] was ameliorated by tolvaptan’s diuretic effect. We acknowledge the likelihood that an increase in renal blood flow may be caused by the diuretic effect of tolvaptan in cases in which the effect was not obtained from diuretics such as furosemide [13]. The effect and mechanism of action of tolvaptan in the maintenance of renal function need to be elucidated.

Vasopressin concentrations were not measured in this study, but it is assumed that they were high [14]. Further, although our patients were in a state of renal failure, it is inferred that some had collecting tubules that were responsive to vasopressin. If this collecting tubule function was measured and evaluated initially, it would have been possible to ascertain whether tolvaptan is effective in disorders such as heart failure with advanced renal failure.

In summary, we examined the additive effect of tolvaptan among patients using other diuretics for severe CKD complicated by congestive heart failure. Urine volume and urine osmolality changed significantly, free water clearance showed a tendency to increase, and tolvaptan showed a consistent effect. Hypernatremia did not occur. There was no exacerbation of the serum Cr level and no adverse effect on renal function. We showed a decrease in the serum Cr level in patients with stage 5 CKD. Tolvaptan is an optional effective diuretic for patients with CKD.
